# The INTErGenerational intervention to Reduce fraIlTY (INTEGRITY) trial

**DOI:** 10.1002/alz70860_102291

**Published:** 2025-12-23

**Authors:** Mei Ling Lim, Amy Sparks, Nicole Ee, Sarah Kedwell, Ebony Lewis, Mao Ling Lim, April Mallon, Christina Coleman, Catherine Ho, Amelia Trippas, Rachelle Scott, Craig S. Anderson, Kim Delbaere, Hiroko H Dodge, Anneke Fitzgerald, Ruth E Hubbard, Monika Janda, Eva Kimonis, Susan Kurrle, Kenneth Rockwood, Stephanie Alison Ward, Craig L Segaert, Ruth Peters

**Affiliations:** ^1^ The George Institute for Global Health, Sydney, NSW, Australia; ^2^ University of New South Wales, Sydney, NSW, Australia; ^3^ Neuroscience Research Australia, Sydney, NSW, Australia; ^4^ Harvard Medical School, Boston, MA, USA; ^5^ Griffith University, Brisbane, QLD, Australia; ^6^ University of Queensland, Brisbane, Australia; ^7^ University of Queensland, Brisbane, QLD, Australia; ^8^ University of Sydney, Sydney, NSW, Australia; ^9^ Dalhousie University, Halifax, NS, Canada; ^10^ Community lead, Sydney, NSW, Australia; ^11^ The George Institute for Global Health, University of New South Wales, Imperial College London, Sydney, NSW, Australia

## Abstract

**Background:**

Programs which facilitate purposeful intergenerational interactions are promising risk‐reduction tools. They can combine strong social engagement alongside cognitive and physical activities targeting executive function, memory and processing speed, moderate intensity movement and balance, themed around key areas e.g. healthy diet to target risk‐reduction for dementia and frailty. However, implementing risk‐reduction opportunities can be challenging, and sustained benefit likely requires long‐term lifestyle change. Intergenerational‐practice represents a unique opportunity to deliver a community‐embedded social and multidomain approach with additional potential benefits for children. However, there is a lack of clinical trial data on effectiveness, particularly outside the United States

**Method:**

The INTEGRITY trial is an on‐going cluster‐randomised wait‐list controlled trial in Sydney, Australia, measuring the impact of multidomain intergenerational‐practice sessions on reducing frailty in older adults. Secondary endpoints include cognition. The intervention consists of weekly 2‐hour sessions conducted in community‐based preschools and involving ∼10 older adults and ∼10 preschool children per site over a 20‐week program. The unit of randomization is the preschool. Outcome assessments for older adults include cognitive and physical ability, mood, quality of life, social connections, loneliness, sleep and the frailty index. Children are engaged from the local preschool and receive assessments on language and empathy. Process evaluation data to assess feasibility and engagement is also collected. The control group receive the same sessions after a 20‐week waitlist period has elapsed

**Result:**

Recruitment and sessions are underway. Over 550 older adults have expressed interest with 317 progressing to screening. Screen success rates are 81.1%. A total of 237 older adults have been allocated to sites and 9 sites (out of a planned 44) have completed their sessions. Adult withdrawal rates are 25% overall and 13.7% for those who start attending sessions, primarily due to ill health (their own/their family). Child recruitment has been similarly successful. Anecdotal feedback from older adults, parents and preschool educators is highly positive, with several preschools requesting repeat sessions.

**Conclusion:**

Preliminary findings suggest intergenerational‐practice is well received and a feasible way to deliver community‐based interventions. Further data will give insight into the potential cognitive, physical and social benefits for older adults and developmental benefits for children.

